# Feuerstein Instrumental Enrichment Program for People With Schizophrenia After the First Episode of Psychosis: Protocol for an Open-Label Intervention Study

**DOI:** 10.2196/57031

**Published:** 2024-09-06

**Authors:** Ana Olivia Fonseca, July Silveira Gomes, Rafael Angulo Condoretti Barros Novaes, Cíntia Lopes Dias, Maria Eva de Miranda Alves Rodrigues, Ary Gadelha, Cristiano Noto

**Affiliations:** 1 First Episode Program Psychiatric Department Federal University of Sao Paulo Sao Paulo Brazil; 2 Clinical Neuroscience Lab Federal University of Sao Paulo Sao Paulo Brazil; 3 Psychobiology Department Federal University of Sao Paulo Sao Paulo Brazil; 4 Clinical Neuroscience Lab Federal University of Sao Paulo Federal University of Sao Paulo Sao Paulo Brazil

**Keywords:** schizophrenia, Feuerstein Instrumental Enrichment program, cognitive intervention, functionality, first-episode psychosis, early stages, Feuerstein Instrumental Enrichment, FIE, psychotic disorder, psychotic disorders, neurocognitive deficits, economic burden, cognitive rehabilitation, functionality, quality of life, daily living, cognitive assessment, maze task, mental disorder, cognitive deficits, mental health, psychosis

## Abstract

**Background:**

Schizophrenia is a disorder associated with neurocognitive deficits that adversely affect daily functioning and impose an economic burden. Cognitive rehabilitation interventions, particularly during the early phases of illness, have been shown to improve cognition, functionality, and quality of life. The Feuerstein Instrumental Enrichment (FIE) program, based on the Mediated Learning Experience and the Structural Cognitive Modifiability theory, has been applied in various disorders, but its applicability in schizophrenia has not yet been clarified.

**Objective:**

This study aims to investigate the effects of the FIE program on the functionality of patients with first-episode schizophrenia.

**Methods:**

In total, 17 patients will be recruited for an open-label intervention consisting of twice-weekly sessions for 10 weeks. The primary outcome measure will be changes in the Goal Achievement Scale score. Maze task performance from the Measurement and Treatment Research to Improve Cognition in Schizophrenia (MATRICS) battery will serve as a secondary outcome measure. At the same time, changes in Positive and Negative Syndrome Scale scores and other MATRICS domains will be analyzed as exploratory outcomes. Assessments will be administered before and after the intervention, with a follow-up period of 6 months.

**Results:**

This trial was preregistered in The Brazilian Registry of Clinical Trials (RBR-4gzhy4s). By February 2024, 11 participants were enrolled in the training. Recruitment is expected to be completed by May 2024. Data analysis will be conducted between May and September 2024. The results are expected to be published in January 2025.

**Conclusions:**

This study may establish a protocol for the FIE program that uses mediation techniques for individuals in the early stages of schizophrenia. The results will add to the knowledge about strategies to promote cognitive skills and functional impairment in daily life.

**International Registered Report Identifier (IRRID):**

DERR1-10.2196/57031

## Introduction

### Background

Schizophrenia is a debilitating mental disorder characterized by hallucinations and disorganized thinking. It is influenced by genetic, neurological, and social factors that affect brain development and functioning [1]. Individuals often exhibit socialization difficulties and cognitive deficits [2] that are associated with low recovery rates [3-6].

Cognitive impairments, including deficits in executive functioning, processing speed, cognitive control, attention, working memory, and social cognition, are core features of schizophrenia [7-9]. Visual processing and perception impairments, including motion, color perception, and perceptual organization, are also observed and are associated with disorganization symptoms [10].

Cognitive deficits can be detected during the early stages of the illness, even up to a decade before the first episode of psychosis [2]. Despite initial improvement [11], the cognitive abilities of individuals experiencing their first episode of psychosis are similar to those of patients with chronic schizophrenia [12-14]. Furthermore, up to 90% of individuals diagnosed with schizophrenia may experience recurrence within 3 years [13]. The connection between clinical and cognitive insight, which in turn affects relapse rates, is well-established [15]. Specific cognitive skills, such as prospective memory, attention, and working memory, have been shown to impact both medication adherence [15] and overall functionality [16].

Cognitive remediation interventions in schizophrenia have been investigated and shown to improve neurocognition [17], although there is limited evidence of the generalization of trained abilities to daily life functioning [18]. On the other hand, studies have shown that cognitive remediation interventions that incorporate psychotherapy features, where therapists actively discuss behavioral and cognitive alternatives for the activities and personalize and contextualize the exercises to the real world, have the potential for higher generalization of cognitive gains to daily life [17,18].

The Feuerstein Instrumental Enrichment (FIE) program [19] is a paper-pencil set of tasks developed by Reuven Feuerstein based on 2 main principles that are, Mediated Learning Experience (MLE) and Structural Cognitive Modifiability (SCM) theory [20,21]. It has 14 tools or instruments, divided into 3 modules according to task complexity and progressive learning skills.

The FIE method is grounded in the foundational principle of altruism, with the intention of providing aid and support to individuals undergoing cognitive transformation. The mediator assumes the responsibility for making activities, tasks, and the environment comprehensible from an individual’s perspective [22]. In the learning process, the mediator has a pivotal role in establishing purposeful intentions, providing guidance, and interpreting and personalizing stimuli to improve an individual’s understanding of the world and themselves. This method fosters metacognition and the acquisition of personal knowledge [23,24].

The MLE is the basis for cognitive modification proposed by the SCM theory [24]. It emphasizes the possibility of developing new behaviors, skills, and cognitive abilities not previously presented in someone’s individual functioning [22]. This repertoire is associated with neuroplasticity and, consequently, neurophysiological changes [22].

SCM theory proposes cognitive modification using MLE [24]. MLE facilitates the acquisition of new behaviors, skills, and cognitive abilities that were not previously present in an individual’s functioning [22]. This acquisition is associated with neuroplasticity and subsequent neurophysiological changes [22]. The FIE program, a top-down paradigm, has been previously used for visual-perception organization training in patients with schizophrenia [25]. It has been associated with improvements in perceptual organization abilities and broader cognitive functions compared with computer-based bottom-up interventions such as CogPack (marker software GmbH).

Instrumental enrichment has been previously used for visual-perception organization training in patients with schizophrenia by Kurylo et al [25]. They compared 2 interventions, focused on perceptual organization, to a control intervention (a computer nonvisual-perceptual task, CogPack), of which one was a computer-based bottom-up intervention based on Gestalt principles, requesting the participant to group stimuli according to visual characteristics, like luminance, color, line orientation, and motion. The other was 2 of the 14 instruments from the FIE program, following a top-down paradigm: organization of dots and an analytic perception task. They found that both visual perception interventions were superior to the control task for the improvement of perceptual organization ability, generalizing gains to other visual cognitive tasks. Furthermore, FIE was also associated with improvements in broader cognitive functions compared with the other 2 interventions.

While FIE has the potential to improve cognitive flexibility and problem-solving through group mediation, its potential to generalize has not been explored. Therefore, this study aims to investigate the effects of peer-mediated FIE interventions on daily life functionality in patients with FES. The hypothesis is that FIE intervention will lead to improvements in daily life functionality in patients with FES.

Regardless of the cognitive visuo-perceptual improvement, FIE has great potential to improve cognitive flexibility and problem-solving through group mediation and has a high potential to generalize 2 features that were not explored by Kurylo et al [25]. In this study, we investigate the effects of peer FIE intervention on the functionality of patients with FES. Our main hypothesis is that FIE intervention improves daily life functionality in patients with FES.

### Aims and Objectives

This study aims to investigate the effects of the FIE intervention, delivered through a peer-mediator group format, on functionality in individuals with schizophrenia. Functionality will be evaluated based on individual goal achievement using the Goal Achievement Scale (GAS) [26,27]. Secondary outcomes will include changes in problem-solving and reasoning, measured through the maze task from the Measurement and Treatment Research to Improve Cognition in Schizophrenia (MATRICS) score [28]. Exploratory analysis will also investigate changes in the Positive and Negative Syndrome Scale (PANSS) scores [29] and other MATRICS domains.

## Methods

### Research Design

The proposed research design is an open-label interventional pilot study that aims to investigate the effects of the FIE program on the functionality of patients with FES based on their personal goals. The study will measure outcomes at 3 time points that are baseline (t0), after the intervention (t1), and at the 6-month follow-up (t2).

### Participants

**Figure 1:**
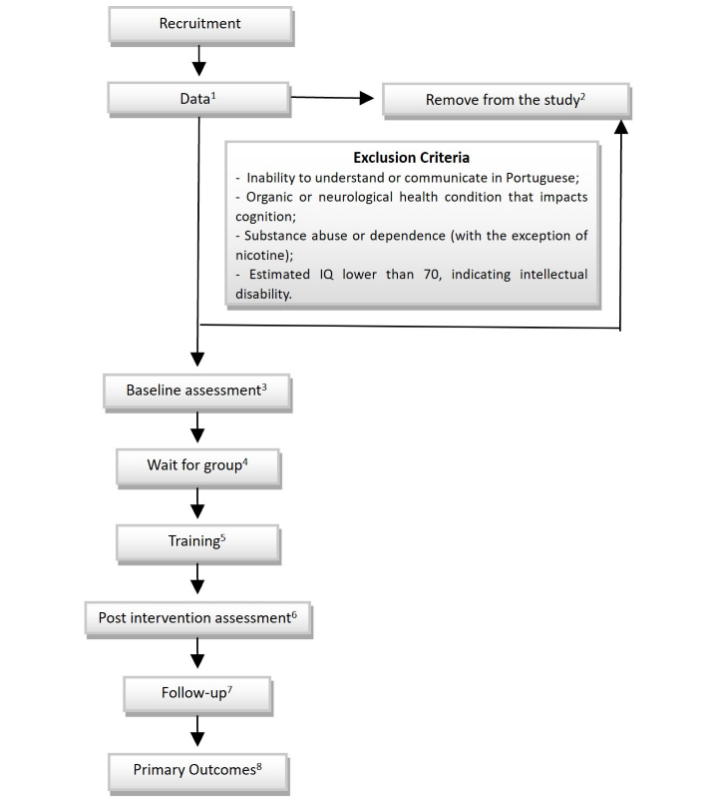
Study design and patient flowchart. (1) We applied the informed consent form, IQ assessment, and Structured Clinical Interview for DSM-5-Research Version. (2) Participants who are deemed ineligible or unwilling to participate or continue in this study will be removed from the study. (3) We conducted baseline assessments using Measurement and Treatment Research to Improve Cognition in Schizophrenia (MATRICS), Positive and Negative Syndrome Scale, and Goal Achievement Scale. (4) We are awaiting a group of 2-3 eligible participants who have successfully completed the IQ and MATRICS assessments. (5) A 10-week training program with Feuerstein Instrumental Enrichment (FIE) will be conducted, with a frequency of 2 sessions per week. (6) A postintervention assessment will be conducted 1 week after the completion of the FIE program. (7) A follow-up assessment will be conducted 6 months after the completion of the FIE program. (8) The primary outcome of the study will be functionality, which will be measured using the Goal Attainment Scale scores. In addition, problem-solving and reasoning abilities will be assessed using the maze scores from the MATRICS assessment.

Participants for this study will be recruited from the Early Intervention Group of the Federal University of São Paulo, as well as from other first-episode programs and public and private psychiatric offices in São Paulo. To be eligible, participants must be between 18 and 40 years old and have a diagnosis of schizophrenia according to the *Diagnostic and Statistical Manual of Mental Disorders, Fifth Edition* (*DSM-5*) criteria [30], as confirmed by the Structured Clinical Interview for DSM-5-Research Version (SCID-5 RV) Axis I disorders [31], and received treatment for psychosis while being clinically stable, according to their psychiatrist. Participants must also have an estimated IQ of 70 or higher and be able to provide informed consent. Exclusion criteria include an inability to understand or communicate in Portuguese, any organic or neurological health condition that impacts cognition, substance abuse or dependence (except nicotine), and an estimated IQ lower than 70, indicating intellectual disability. A patient flowchart is shown in Figure 1.

### Intervention

The intervention will comprise 2 weekly sessions conducted over a period of 10 weeks. Patients will be enrolled in a pair or 3-person group and will always be accompanied by a mediator. Participation will be continuous and dependent on the patients’ interest and fulfillment of the inclusion criteria. The intervention will use 3 tasks from the FIE program-standard instrument I, namely organization of dots, orientation in space I, and analytic perception [32].

The organization of dots task involves identifying geometric images from a cloud of dots; the patient is required to draw lines to connect the dots and form the geometric image. To complete this task, it is necessary to project and create a virtual relationship between elements, focusing on the relevant aspects of the figure.

The orientation in space I task focuses on visuospatial flexibility and perception of the world, making the patient recognize and differentiate the position of objects on the paper sheet, exploring the patient’s own references regarding left, right, front, and back to identify the position of objects in relation to others. This task promotes problem definition and solving strategies, hypothetical thinking, and the creation of conditionals (ie, If… then...) and comparison strategies with peers.

The analytic perception task practices the differentiation and integration of images. In this task, there is a model image, and there are several options for possible responses. The options are a set of shapes that need to be mentally grouped to form the model image, such as a jigsaw. The patient needs to analyze the options and choose the 1 that corresponds to the model. It explores the perceptual ability to focus on specificities, splitting the whole into parts, and the ability to see the whole picture, gathering parts into 1 element.

Each session will take 1-hour and involves 3 steps [20,32]:

1. Introduction: The mediator highlights the task’s novelty and complexity, guiding patients toward the solution to the problem.
2. The task itself, where patients will work with the instrument and mediator, will observe their behavior, strategies, and reactions, mediating the process and acting as a bridge between the instrument and patients. The mediator will also provide feedback and correct answers and prevent frustration, among other things.
3. Conclusion: Patients will practice the transfer of knowledge acquired during the session to daily life, discussing the exercise’s realization and the processes used during the intervention.

In this pilot study, one of the coauthors (MEDMAR) will be enrolled as a mediator, applying the intervention to all participants in all groups. Furthermore, 2 parallel groups can be run over time. The mediator graduated in the educational field and has been certified by the Feuerstein Institute since 2009. She has more than 10 years of experience as an FEI practitioner and is currently an authorized FEI trainer in Brazil.

### Outcomes

The primary objective is to assess the impact of the intervention on functionality, as measured by the GAS [26,27,33], which evaluates the extent to which participants achieve their individual goals. The secondary objective is to evaluate problem-solving and reasoning skills using the maze score from the MATRICS assessment [28]. An exploratory analysis will also examine changes in PANSS scores and other domains of the MATRICS assessment. All assessments will be conducted at the end of the 10-week intervention period and during the 6-month follow-up period.

### Clinical Assessments

To evaluate clinical symptomatology, the PANSS will be used [34]. PANSS is a widely used instrument consisting of 30 items and is used to measure the severity of clinical symptoms in patients with schizophrenia. The interviewee psychiatrist is rated on a scale of 1 to 7 by the interviewer psychiatrist.

### Functionality Assessments

The GAS is a type of assessment that is centered on personal goals and is commonly used in physical rehabilitation (lifestyle, type of handicap, and aspirations) but is not often used in psychiatry [26,27]. GAS was first used in mental health settings in the 1960s and has also been applied for cognitive rehabilitation [33,35].

The patient and interviewer work together to define individual goals that are specific, measurable, attainable, realistic, and timely, using the “SMART” method. These goals are based on the patient’s current lifestyle, daily routines, interests, and needs. If a patient expresses a desire for a long-term goal that cannot be achieved in a short time, the interviewer helps the patient break it down into smaller achievable goals that fit within a defined time frame. The GAS score is based on a defined goal and considers its weightage. Achievement of the defined goal is typically scored as 0, and if the achievement is better or worse than that defined, the score is either +1 or +2 or 1 or 2, respectively. The patient also rates the importance and difficulty of each goal on a 0-3 Likert scale. The final score is calculated by an algorithm that provides a baseline score, an achieved score, and the difference between the two [27].

### Cognitive Assessments

The estimated IQ (for inclusion criteria) will be assessed using 2 subtests from the Brazilian version of the Wechsler Intelligence Scale [36], which are vocabulary and rational reasoning.

To assess cognitive changes, 9 tests from the Brazilian version of the MATRICS [28,37] will be used. The test evaluates 7 cognitive domains:

1. Speed of processing: Trail Making Test, Brief Assessment of Cognition in Schizophrenia Symbol Coding, and Category Fluency Animal Naming Test
2. Attention: Continuous Performance Test-Identical Pairs
3. Working memory: Spatial Span and Letter-Number Span Test
4. Verbal learning: Hopkins Verbal Learning Test-Revised
5. Visual learning: Brief Visuospatial Memory Test-Revised.
6. Reasoning and problem-solving: neuropsychological assessment battery maze.

The Mayer-Salovey-Caruso Emotional Intelligence Test Managing Emotions subtest was excluded due to its low-reliability coefficient in Brazilian versions [28].

### Power and Sample Size

Sample calculation was performed using G*Power software (Heinrich-Heine-University), considering a post hoc analysis for 17 participants, effect size (Cohen *d*=0.5) [25], and estimated power (β=.62), where the GAS score means will be compared pre- and post-FIE intervention (described in the Intervention section) using a matched pairs Student *t* test.

### Analysis

Data will be analyzed using IBM SPSS (version 21) or Jamovi software following the intention-to-treat approach. The last observation carried forward will be the procedure to impute the missing data. Sociodemographic and baseline information of the patients will be described to show the sample characteristics.

The study will use ANOVA or Kruskal-Wallis tests to analyze changes in numerical variables, including primary and secondary outcomes, as well as exploratory analyses, depending on the data distribution. Post hoc pairwise comparisons will be conducted using the Bonferroni adjustment for multiple comparisons in the Friedman test. The chi-square test will be used to analyze categorical data, such as sex. The effect size will be calculated using Cohen *d*, based on the mean and SD of the mean t1 for the GAS.

### Ethical Considerations

To ensure that ethical standards were met, the study adhered to the principles of the Declaration of Helsinki and the guidelines of Good Clinical Practice. The Ethics Committee of the Federal University of São Paulo approved the study (A00951289489), and it was preregistered in The Brazilian Registry of Clinical Trials (RBR-4gzhy4s). Participation will be voluntary and not remunerated, and the participants will only be enrolled after providing informed consent, which has been approved by the Ethics Committee. All patient data will be safeguarded by the research team to maintain participant anonymity and confidentiality.

## Results

This trial has been preregistered in the Brazilian Registry of Clinical Trials (RBR-4gzhy4s). Recruitment started in February 2022, and by February 2024, 11 participants were enrolled in the training. Recruitment is expected to be completed by August 2024. Data analysis will be conducted between May and September 2024. The results are expected to be published in peer-reviewed journals and presented at mental health conferences after January 2025.

## Discussion

### Principal Findings

The FIE program is an ecological intervention that requires patients to apply strategies to solve tasks and generalize them to their daily lives. By promoting neuroplasticity, cognitive flexibility, self-awareness, and socialization, our study is designed to offer space for patients to engage in productive discussion and socialization through mediation, which may enhance patient insight into their own cognitive processes. The FIE program allows patients to build knowledge by sharing ideas with peers and mediators [21], which may improve engagement and self-esteem. In addition, cognitive abilities such as attention, visual perception, organization, and flexibility are required to perform these activities [25]. Under this intervention, we aim to challenge patients in a safe environment, creating opportunities for the generalization of real-life strategies.

The GAS assessment is a valuable tool not only for project staff to report changes in functionality but also for the patients themselves. By tracking the patient’s progress toward their own goals, regardless of cognitive changes, they can become active participants in their own recovery process. This helps to increase motivation, engagement, and self-esteem and ultimately leads to better treatment outcomes. Our goal of evaluating functionality as a primary outcome was to focus on recovery from the patient’s perspective, rather than data change, as an isolated construct.

The early years following the first episode of schizophrenia are crucial for recovery. Antipsychotic medications can improve positive symptoms, however, adherence to treatment may be highly influenced by psychological and behavioral interventions that aim to enhance insight and neuroplasticity. In addition to understanding the nature of the illness, it is important for individuals with schizophrenia to gain insight into their functional and cognitive deficits as part of the recovery process. This can help develop realistic goals and strategies to improve their overall functioning and quality of life. By recognizing and addressing these deficits, individuals with schizophrenia can play a more active role in their recovery and work toward achieving their full potential.

Professional capacitation in FIE requires intense training of the mediator, who needs to go through the experience of being mediated before becoming a mediator, using an immersive approach. Some of the FIE assumptions, such as intentionality or reciprocity and the mediation of transcendence, focus the interactions on the needs of the patient and on learning as a process that transcends the task itself. The mediator’s intentionality involves ensuring the task and the information can be effectively understood by the patient, shaping the behavior and experience to a more holistic outcome [38]. Having a person with this profile as part of a multidisciplinary team can enrich the interaction of the professional group, expanding the perspective of how the team works with the patient and the patient himself translating the knowledge for daily life behavior for the group. After having a professional trained in FIE, it becomes an accessible and low-cost intervention.

Considering the topics described above, it is necessary to explore the effects of this intervention and disseminate the results to a broader community. Given the high prevalence of schizophrenia and its associated functional impairments, comprehensive interventions such as FIE may have positive impacts on the recovery process, which are essential public health priorities, particularly in low- and middle-income countries, where access to effective treatment is often limited. If this study shows a gain in functionality, the dissemination of this technique can increase access to evidence-based treatments, ultimately leading to improved outcomes for individuals with schizophrenia.

### Limitations

The major limitation of this study is the absence of a control group, which was a decision made due to several issues recognized by the research team, including sample characteristics and the onset of the SARS-CoV-2 pandemic. The recruitment of patients after their first episode of schizophrenia within a 5-year time window posed a challenge, as many patients may have a long duration of untreated psychosis or have already had the disease for more than 5 years. In addition, some patients in remission may decline to participate in the intervention for personal reasons, such as a lack of insight into the disease or the side effects of medication, both of which can affect the willingness to participate in psychosocial interventions.

Other limitations are associated with the FIE technique. The mediator must be a specialist in the technique and trained by an authorized mentor or institution. Training a mediator in FIE is challenging not only because of its cost of nearly US $1000, equivalent to 72 hours of initial formation but also because of the need for a mediator’s constant practice in order to develop the required skills that will enable him or her to work with the sample while maintaining the focus on the FIE proposition. Nowadays, this may represent a barrier to replicating the methodology in the general medical system. However, other behavioral techniques faced the same challenges when they were first applied. An example of this was the challenges faced by applied behavior analysis (ABA) applied to autism in the 60th/70th [39]. To be an ABA specialist, someone must be trained for approximately 2000 hours [40]. In addition, the gains achieved by the patients made it the first line of intervention for this population, leading to ABA popularization [39]. Therefore, difficulties in FIE capacitation and training may exist but should not discourage the investigation of its effects on schizophrenia.

Another possible issue is patient resistance to attending the intervention twice per week. We will provide a friendly and welcoming environment for intervention and rely on the possible gains as a key to maintaining adherence and reducing dropouts. Furthermore, none of the 11 participants enrolled by now dropped out of the study.

### Conclusions

In conclusion, our project focuses on the investigation of the effects of the FIE program in patients with first-episode schizophrenia. Our results may show if this ecological approach, focused on generating insight and neuroplasticity, will impact functionality. If so, we expect to help patients become active participants in their own recovery process. Despite the limitations of this study, such as the absence of a control group and challenges associated with professional FIE training, investigating FIE intervention in first-episode schizophrenia is valuable because of the low cost of application and accessibility of this technique. Furthermore, we are exploring some gaps that still exist in the treatment of people with schizophrenia: (1) there is no effective intervention recognized as a first line to improve cognition and functionality in schizophrenia; (2) the current cognitive intervention programs in schizophrenia yield few or no effective generalizations to daily life; and (3) the importance of assessing functionality based on the patient’s own goal, as we will do with the GAS, instead of relying only on scale scores. Our research can contribute to increasing the knowledge of nonpharmacological interventions for schizophrenia.

## Abbreviations

ABA: applied behavior analysis
*DSM-5*: *Diagnostic and Statistical Manual of Mental Disorders, Fifth Edition*
FES: first-episode schizophrenia
FIE: Feuerstein Instrumental Enrichment
GAS: Goal Achievement Scale
MATRICS: Measurement and Treatment Research to Improve Cognition in Schizophrenia
MLE: mediated learning experience
PANSS: Positive and Negative Syndrome Scale
SCID-5 RV: Structured Clinical Interview for DSM-5-Research Version
SCM: Structural Cognitive Modifiability

## Data Availability

Due to privacy restrictions, participants’ previous consent did not include raw data, and therefore, these sets will not be deposited in publicly available repositories. Thus, in order to have access to any data besides those presented in the results session, a request must be submitted by email to the corresponding author, who will only grant access to those data if possible and according to the Ethics Committee of the Federal University of São Paulo approved considerations.
